# Supplementary material for: A large gene family in fission yeast encodes spore killers that subvert Mendel’s law
Source: eLife. 2017 Jun 20;6:e26057. doi: 10.7554/eLife.26057 (PMC5478263; doi:10.7554/eLife.26057)
Supplement: Supplementary file 2 — DOI: http://dx.doi.org/10.7554/eLife.26057.026 [file elife-26057-supp2.doc]

Supplementary File 2. Fission yeast strains used in this study

| Name | Background | Mating  type | Genotype | Use |
| --- | --- | --- | --- | --- |
| LD775 | Lab strain | h- | *leu1-32* | Figure 1-S1 |
| DY8531 | Lab strain | h- | *ura4-D18 leu1-32 inversion_junction_1::Padh1-kanMX inversion_junction_2::ura4+* | Figure 1A and 1-S1 |
| DY9974 | CBS5557 | h+ | *mat1PΔ17::natMX* | Figure 1A and 1-S1 |
| DY26097 | Hybrid of lab strain and backcrossed-1 | h+/h- | *mug123Δ::kanMX/mug123+ leu1-32/leu1-32 lys1+/lys1-131 ade6+/ade6-M216 ura4+/ura4-D18 mat1PΔ17::bsdMX/mat1MΔ17::natMX* | Figure 1B |
| DY26095 | Hybrid of lab strain and backcrossed-2 | h+/h- | *rps2802Δ::kanMX/rps2802+ leu1-32/leu1-32 lys1+/lys1-131 ade6+/ade6-M216 ura4+/ura4-D18 mat1PΔ17::bsdMX/mat1MΔ17::natMX* | Figure 1C |
| DY26100 | Hybrid of lab strain and backcrossed-1 | h+/h- | *cw9Δ::hphMX* in DY26097 | Figure 1B |
| DY26092 | Hybrid of lab strain and backcrossed-2 | h+/h- | *cw27Δ::hphMX* in DY26095 | Figure 1C |
| DY21782 | CBS5557 | h+/h- | *mat1PΔ17::natMX/mat1MΔ17::bsdMX* | Figure 2A and 2-S1 |
| DY21838 | CBS5557 | h+/h- | *cw9Δ::kanMX/cw9Δ::kanMX mat1PΔ17::natMX/mat1MΔ17::bsdMX* | Figure 2A and 2-S1 |
| DY21842 | CBS5557 | h+/h- | *cw27Δ::hphMX/cw27Δ::hphMX mat1PΔ17::natMX/mat1MΔ17::bsdMX* | Figure 2A and 2-S1 |
| DY21547 | CBS5557 | h+/h- | *cw9Δ::kanMX/cw9+ mat1PΔ17::natMX/mat1MΔ17::bsdMX* | Figure 2A and 2B |
| DY21550 | CBS5557 | h+/h- | *cw27Δ::hphMX/cw27+ mat1PΔ17::natMX/mat1MΔ17::bsdMX* | Figure 2A and 2B |
| DY34484 | CBS5557 | h+/h- | *cw27Δ::hphMX/cw27+ cw9+/cw9Δ::kanMX mat1PΔ17::natMX/mat1MΔ17::bsdMX* | Figure 2A and 2B |
| DY23132 | Lab strain | h+/h- | *his3Δ::cw27(kanMX)/his3+ leu1+/leu1-32 lys1+/lys1-131 ade6+/ade6-M216 ura4+/ura4-D18 mat1PΔ17::bsdMX/mat1MΔ17::natMX* | Figure 3A and 3-S1 |
| DY23138 | Lab strain | h+/h- | *his3Δ::vector(kanMX)/his3+ leu1+/leu1-32 lys1+/lys1-131 ade6+/ade6-M216 ura4+/ura4-D18 mat1PΔ17::bsdMX/mat1MΔ17::natMX* | Figure 3A and 3-S1 |
| DY26806 | Lab strain | h+/h- | *lys1Δ::cw9(kanMX)/lys1-131 leu1+/leu1-32 ade6+/ade6-M216 ura4+/ura4-D18 mat1PΔ17::bsdMX/mat1MΔ17::natMX* | Figure 3A and 3-S1 |
| DY21463 | Lab strain | h- | *leu1-32 lys1-131 ade6-M216 ura4-D18 mat1MΔ17::natMX* | Figure 3A and 4A, 3-S1, and 4-S2 |
| DY35139 | Lab strain | h+ | *leu1-32::cw9(leu1+) lys1-131 ade6-M216 ura4-D18 mat1PΔ17::bsdMX* | Figure 3A, 4A, 3-S1, and 4-S2 |
| DY34668 | Lab strain | h+ | *leu1-32::cw27(leu1+) lys1-131 ade6-M216 ura4-D18 mat1PΔ17::bsdMX* | Figure 3A, 4A, 3-S1, and 4-S2 |
| DY35067 | Lab strain | h+/h- | *leu1-32::cw27(leu1+)-linked-kanMX/leu1-32::cw9(leu1+)-linked-hphMX lys1-131/lys1+ ade6-M216/ade6-M216 ura4-D18/ura4-D18 mat1PΔ17::bsdMX/mat1MΔ17::natMX* | Figure 3B and 3-S2 |
| DY35268 | Lab strain | h+/h- | *leu1-32::cw9(leu1+)-linked-kanMX/leu1-32::cw9(leu1+)-linked-hphMX lys1-131/lys1+ ade6-M216/ade6-M216  ura4-D18/ura4-D18 mat1PΔ17::bsdMX/mat1MΔ17::natMX* | Figure 3B and 3-S2 |
| DY34998 | Lab strain | h+/h- | *leu1-32::cw27(leu1+)-linked-kanMX/leu1-32::cw27(leu1+)-linked-hphMX lys1-131/lys1+ ade6-M216/ade6-M216 ura4-D18/ura4-D18 mat1PΔ17::bsdMX/mat1MΔ17::natMX* | Figure 3B and 3-S2 |
| DY35066 | Lab strain | h+/h- | *leu1-32::pDUAL-vector(leu1+)-linked-kanMX/leu1-32::pDUAL-vector(leu1+)-linked-hphMX lys1-131/lys1+ ade6-M216/ade6-M216 ura4-D18/ura4-D18 mat1PΔ17::bsdMX/mat1MΔ17::natMX* | Figure 3B and 3-S2 |
| DY34850 | Lab strain | h+ | *leu1-32::cw9-Ta(leu1+) lys1-131 ade6-M216 ura4-D18 mat1PΔ17::bsdMX* | Figure 4A and 4-S2 |
| DY34852 | Lab strain | h+ | *leu1-32::cw9-Tb(leu1+) lys1-131 ade6-M216 ura4-D18 mat1PΔ17::bsdMX* | Figure 4A and 4-S2 |
| DY34854 | Lab strain | h+ | *leu1-32:cw9-Tc(leu1+) lys1-131 ade6-M216 ura4-D18 mat1PΔ17::bsdMX* | Figure 4A, 4B, 4-S2, and 4-S3 |
| DY34856 | Lab strain | h+ | *leu1-32::cw9-Td(leu1+) lys1-131 ade6-M216 ura4-D18 mat1PΔ17::bsdMX* | Figure 4A and 4-S2 |
| DY35478 | Lab strain | h+ | *leu1-32::cw9-Te(leu1+) lys1-131 ade6-M216 ura4-D18 mat1PΔ17::bsdMX* | Figure 4A and 4-S2 |
| DY34840 | Lab strain | h+ | *leu1-32::cw27-Ta(leu1+) lys1-131 ade6-M216 ura4-D18 mat1PΔ17::bsdMX* | Figure 4A and 4-S2 |
| DY34842 | Lab strain | h+ | *leu1-32::cw27-Tb(leu1+) lys1-131 ade6-M216 ura4-D18 mat1PΔ17::bsdMX* | Figure 4A and 4-S2 |
| DY35012 | Lab strain | h+ | *leu1-32::cw27-Te(leu1+) lys1-131 ade6-M216 ura4-D18 mat1PΔ17::bsdMX* | Figure 4A and 4-S2 |
| DY34844 | Lab strain | h+ | *leu1-32::cw27-Tc(leu1+) lys1-131 ade6-M216 ura4-D18 mat1PΔ17::bsdMX* | Figure 4A, 4B, 4-S2, and 4-S3 |
| DY34994 | Lab strain | h+ | *leu1-32::cw27-Td(leu1+) lys1-131 ade6-M216 ura4-D18 mat1PΔ17::bsdMX* | Figure 4A and 4-S2 |
| DY35163 | Lab strain | h- | *leu1-32 lys1-131 ade6-M216 ura4-D18 ars1::cw27-Td(ura4+) mat1MΔ17::natMX* | Figure 4B and 4-S3 |
| DY35165 | Lab strain | h- | *leu1-32 lys1-131 ade6-M216 ura4-D18 ars1::pDUAL-vector(ura4+) mat1MΔ17::natMX* | Figure 4B and 4-S3 |
| DY35080 | Lab strain | h- | *leu1-32 lys1-131 ade6-M216 ura4-D18 ars1::cw9-Td(ura4+) mat1MΔ17::natMX* | Figure 4B and 4-S3 |
| DY34843 | Lab strain | h+ | *leu1-32::cw27-Tc(leu1+) lys1-131 ade6-M216 ura4-D18 mat1PΔ17::bsdMX* | Figure 4B, 4-S3 |
